# Magnetic and structural data used to monitor the alloying process of mechanically alloyed Fe_80_Ni_20_

**DOI:** 10.1016/j.dib.2018.06.036

**Published:** 2018-06-21

**Authors:** Michael W.R. Volk, Michael R. Wack, Bernd Maier

**Affiliations:** a Institute for Rock Magnetism, Department of Earth Sciences, University of Minnesota, 116 Church Street SE, Minneapolis, MN 55455, United States; b Department of Earth and Environmental Sciences, Ludwig Maximilians Universität, Theresienstr. 41, 80333 Munich, Germany

## Abstract

In the last decades, much attention was given to mechanical alloying as it proved to be a cheap and easy way to produce (even metastable) nanostructured alloys. Especially Fe-Ni alloys have been studied intensely due to their technological and scientific importance. The MA process, however, is not fully understood. Furthermore, remanence properties of Fe_80_Ni_20_ are not well known. In our article “*Monitoring the alloying process of mechanically synthesized Fe*_*80*_*Ni*_*20*_*through changes in magnetic properties* (DOI: j.jallcom.2017.10.090, Volk et al., 2018) [1])” we investigated structural and magnetic properties of the intermediate and final alloys. Elemental Fe (99.5%) and Ni (99.7%) powders were filled in a 80 ml zirconia vials together with 3 mm zirconia milling balls and milled at 400 PRM with a planetary ball mill (Fritsch Pulverisette Premium 7). By subsampling the product at 14 different times during the process, the data presented here shows how crystalline structure (X-ray diffraction) and magnetic properties, induced as well as remanent, of the metastable Fe_80_Ni_20_ change during the mechanical synthesis.

**Specifications Table**

**Table:** 

Subject area	*Material sciences, Earth sciences, Planetary Sciences*
More specific ;subject area	*Mechanical alloying, Mechanical synthesis, Intermetallic alloys, Alloying, Meteorite studies, Magnetic properties of Fe*—*Ni alloys*
Type of data	– *Unprocessed magnetic data (hysteresis, direct current demagnetization and isothermal remanent magnetization acquisition) in Princeton Vibrating Sample Magnetometer(VSM) file format* – *X-ray diffraction xy data*
How data was acquired	XRD:
	STOE Stadi P diffractometer in Debye-Scherrer geometry using Mo-kα_1_ (*λ* = 0.0709 nm)
	Magnetic Measurements:
	Princeton Measurements Corporation model 3900 vibrating sample magnetometer
Data format	*Raw-data and figures*
Experimental factors	*Milling parameters:*
	– *400 RPM* – *Fe = 7.92 g, Ni = 2.08 g* – *100 g, 3 mm Zirconia milling balls* – *80 ml zirconia milling vial*
	*All sample preparation steps (e.g. filling of gel capsules) were done in a glovebox with Argon atmosphere to minimize oxidation. O*_*2*_*levels were monitored constantly (< 2%)*
	*Subsamples were taken after 1, 2, 4, 6, 10, 16, 30, 60, 120, 240, 480, 960, 1440 and 2160 min of milling*
	*XRD:*
	*Powder was measured in* glass capillaries with 100 μm diameter and 10 μm.
	*Magnetic measurements:*
	*approx. 50 mg of sample was measured and filled into a gel-capsule. The remaining space was filled with high purity quartz-wool*
Experimental features	*XRD:*
	– *samples are placed in the capillary* – *measurement is done from 10–70° 2 Theta in 0.15° steps with a measurement time of 360 s per step*
	*Hysteresis:*
	– *Magnetization is measured while magnetic field is ramped from 0 T -> +1.5 T -> -1.5 T -> +1.5 T* – *Magnetization is measured every 1mT with an integration time of 100* *ms, using sweeping mode in the VSM (continuous field sweep compared to discrete steps)*
	*IRM acquisition:*
	– *The sample is AC demagnetized in the VSM prior to the IRM ac. measurement* – *Starting from a demagnetized state increasingly strong* *magnetic fields are applied to the sample. After each field step, the remanent magnetization is measured in the residual field of the VSM* – *The field steps are chosen by the VSM software (logarithmic)* – *Measurement time per point = 1 s, maximum field = 500* *mT*
	*DCD:*
	– *After the IRM measurement the sample acquires a saturating moment in + 1 T* – *Similar to the IRM measurement an increasingly larger negative field is applied and the remaining remanence measured* – *The field steps are chosen by the VSM software (logarithmic)* – *Measurement time per point = 1 s, maximum field = − 500* *mT*
Data source location	
Data accessibility	*With article*
Related research article	*Volk, M.W.R., Wack, M.R., Maier, B. (2017). Monitoring the alloying process of mechanically synthesized Fe80Ni20 through changes in magnetic properties. J. Alloy. Compd. 732 (Suppl. C) 2018, S336-S342*

**Value of the data**

● Detailed structural analysis as well as changes in Magnetic properties during mechanical synthesis is essential for understanding of formation of metastable bcc Fe—Ni alloys (i.e. of Fe_80_Ni_20_).
● Evolution of magnetic properties need to be well characterized so they can be used as a proxy for the homogeneity and alloying status of Fe_80_Ni_20_ and possibly other alloys.
● Remanence properties of most Fe—Ni alloys unknown. But knowledge of the magnetic remanence properties of Fe_80_Ni_20_ is important to our understanding of the magnetic signature of meteorites and early planetesimals recovered from meteorites.

## 1 Data

Mechanical alloying proved to be a simple way to synthesize intermetallic alloys. The data set shows the influence of increasing milling times on magnetic properties determined from magnetic hysteresis, direct current demagnetization (DCD) and isothermal remanent magnetization (IRM) acquisition [1]. Furthermore, the X-ray diffraction patterns of the FeNi_20_ alloy are included, which show the progression of alloying, strain and crystallite size.

## 2 Experimental design, materials, and methods

We used a Fritsch Pulverisette Premium (P7) planetary ball mill to synthesize Fe80Ni20. Iron (99.5%, 7.92 g) and Ni (99.7%, 2.08 g) were filled into an 80-ml zirconia vial together with 100 g 3 mm sized milling balls. The vials were prepared in an Ar-filled glove box (O2 < 2%).

X-ray diffraction was measured with a STOE Stadi P diffractometer in Debye-Scherrer geometry using Mo-kα_1_ (*λ* = 0.0709 nm) from 10° to 70° 2Θ in 0.15° steps (360 s).

**Fig. 1:**
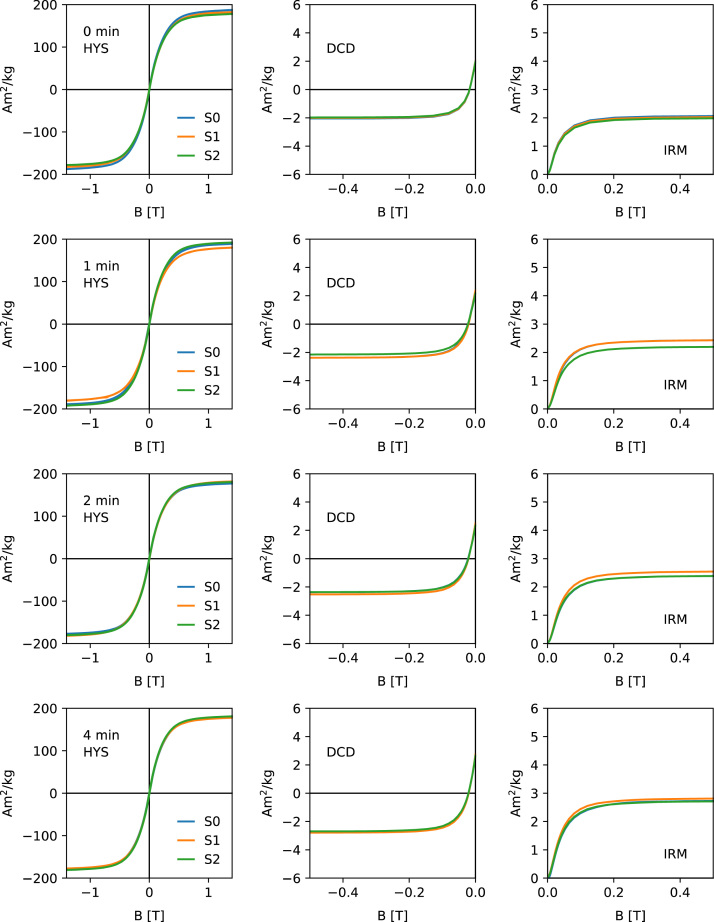
Unprocessed hysteresis loops (first column), direct current demagnetization curves (second column) and acquisition of an isothermal remanent magnetization (right column) for milling times 0–4 min. Data shows the three specimens (S1–S3).

**Fig. 2:**
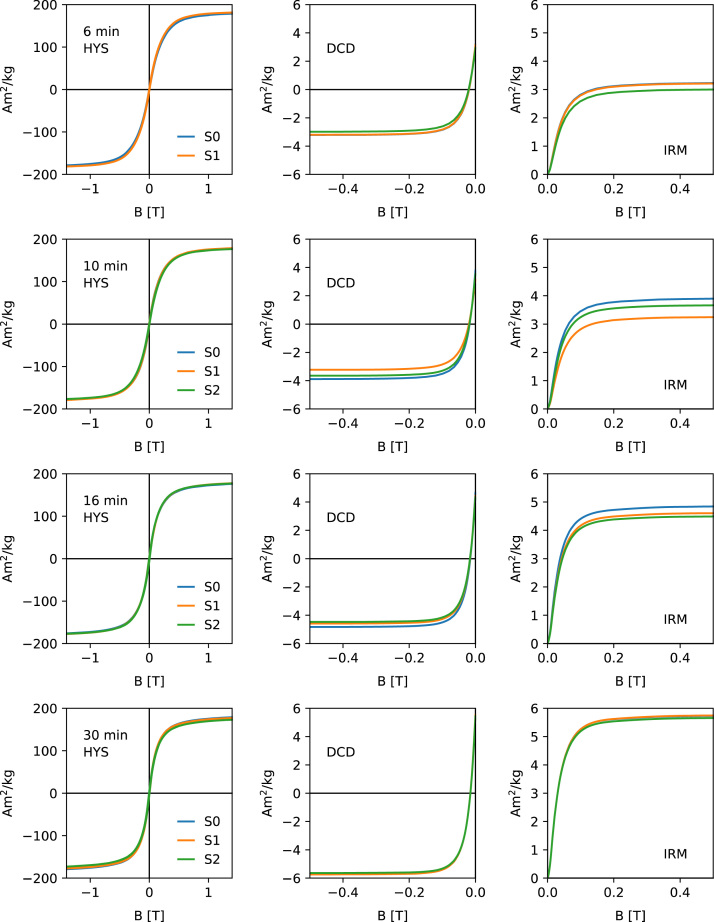
Unprocessed hysteresis loops (first column), direct current demagnetization curves (second column) and acquisition of an isothermal remanent magnetization (right column) for milling times 6–30 min.

**Fig. 3:**
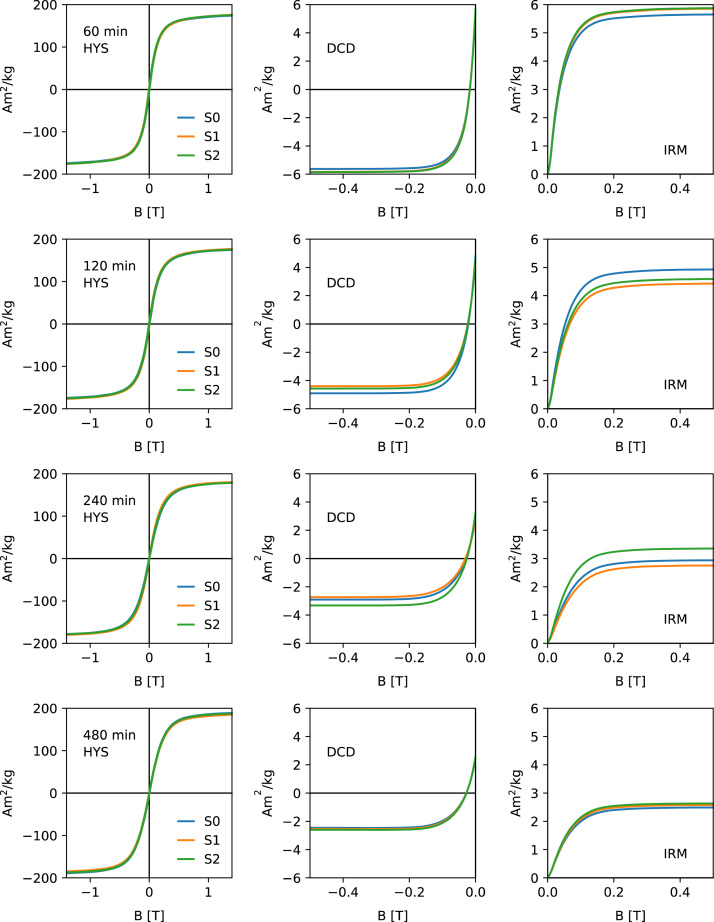
Unprocessed hysteresis loops (first column), direct current demagnetization curves (second column) and acquisition of an isothermal remanent magnetization (right column) for milling times 60–480 min.

**Fig. 4:**
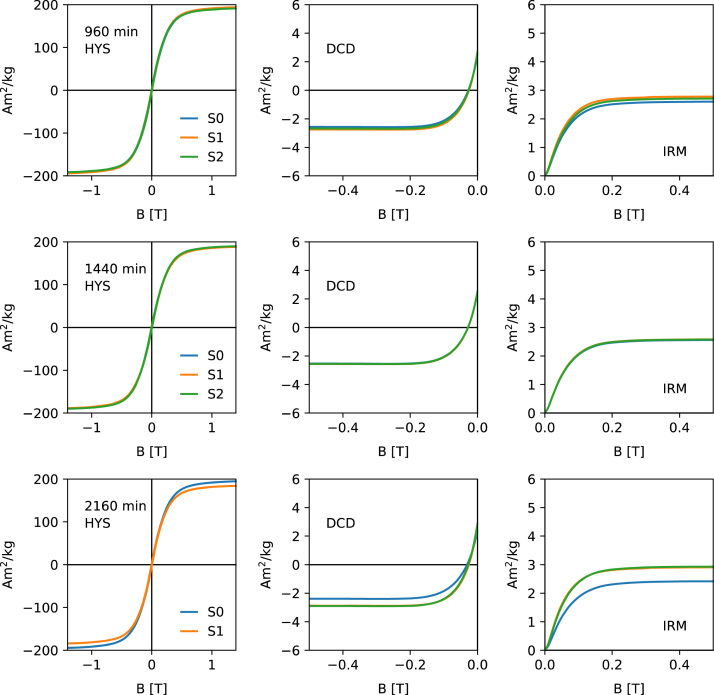
Unprocessed hysteresis loops (first column), direct current demagnetization curves (second column) and acquisition of an isothermal remanent magnetization (right column) for milling times 960–2160 min.

Fig. 1, Fig. 2, Fig. 3, Fig. 4 show the unprocessed Hysteresis (HYS, left column), DCD (middle column) and IRM acquisition curves (right column). For each milling time three specimens were measured. Each specimen was prepared by filling ca. 50 mg of the powder together with quartz wool into a gel-capsule. Subsampling and sample preparation were done inside a glovebox, filled with Ar. The O_2_ concentration was monitored consistently and never exceeded 2%. A Princeton Measurements Corporation model 3900 vibrating sample magnetometer (VSM) was used for the measurements. For each specimen, the same measurement parameters were used. HYS, IRM and DCD were done in sequence.
